# Glucocorticoids in *Systemic Lupus Erythematosus*. Ten Questions and Some Issues

**DOI:** 10.3390/jcm9092709

**Published:** 2020-08-21

**Authors:** Sabrina Porta, Alvaro Danza, Maira Arias Saavedra, Adriana Carlomagno, María Cecilia Goizueta, Florencia Vivero, Guillermo Ruiz-Irastorza

**Affiliations:** 1 Rheumatology Department, Hospital JM Ramos Mejía, Buenos Aires 1221, Argentina; psachu@gmail.com (S.P.); mairaariassaavedra@gmail.com (M.A.S.); 2 Department of Internal Medicine, Faculty of Medicine, Universidad de la República, Montevideo 11000, Uruguay; alvarodanza@gmail.com (A.D.); adrianacarlomagno@gmail.com (A.C.); 3 Autoimmune Disease Unit, Sanatorio 9 de Julio, Tucumán T4000, Argentina; cecigoizueta@yahoo.com.ar; 4 Autoimmune Disease Unit, Hospital Privado de Comunidad, Mar del Plata B7600, Argentina; florenciavivero82@gmail.com; 5 Autoimmune Diseases Research Unit, BioCruces Bizkaia Health Research Institute, Cruces Univeristy Hospital, 48903 Bizkaia, Spain; 6 University of the Basque Country, 48940 Leioa, Spain

**Keywords:** systemic lupus erythematosus, SLE, prednisone, methylprednisolone, glucocorticoids, mortality, prognosis, damage

## Abstract

Since the discovery of glucocorticoids (GCs), their important anti-inflammatory effect, rapid mechanism of action, low cost, and accessibility have made them one of the mainstays of treatment for *Systemic lupus erythematosus* (SLE). Although their use has allowed controlling the disease and reducing acute mortality in severe conditions, the implementation of a scheme based on high doses for long periods has inevitably been accompanied by an increase in adverse effects and infections, including long-term damage. The objective of this review is to answer some important questions that may arise from its use in daily clinical practice, and to propose a paradigm based on the use of methylprednisolone pulses followed by medium-low doses and a rapid decrease of prednisone.

## 1. Introduction

*Systemic lupus erythematosus* (SLE) is a complex disease characterized by autoimmunity, inflammation, and a variable degree of organ damage, which depends on the number and severity of flares but also on the treatments received. The management of SLE is often challenging. Most guidelines refer to “standard of care” as a combination of hydroxychloroquine, glucocorticoids (GCs), and, sometimes, an immunosuppressive agent. Such therapy often achieves disease remission, but too many times at the cost of a large degree of damage accrual.

Irreversible organ damage is not only very frequent in SLE, but also particularly relevant considering that most patients are young or middle-aged women. According to a growing amount of scientific evidence, irreversible damage, as well as other serious side effects, such as infections, are strongly associated with the use of GCs [1,2,3]. Indeed, the recently updated EULAR guidelines highlight the need to prevent organ damage and to optimize pharmacological strategies in order to improve health-related quality of life and to achieve long-term patient survival [4].

The purpose of this review is to answer ten daily clinical practice questions by updating the current evidence about the optimal doses of GCs in several scenarios, based on pharmacological and clinical evidence, as well as to offer our point of view regarding the “standard of care” of GC use.

### 1.1. What Is the Main Mechanism of Action of GCs?

The first mechanism of action of GCs is to interfere with the genomic transcription of inflammatory molecules. This process starts by means of GCs binding the cytosolic-GC receptor (cGR). The GC-cGR complex is translocated into the nucleus where it modulates gene expression. This is called the genomic pathway [5,6]. The first effect generated by the GC-cGR complex within the nucleus is transrepression, consisting of the inhibition of those genes which promote cytokine and other protein synthesis involved in the inflammatory process, with the resulting anti-inflammatory effect. As the intranuclear concentration of GCs increases, a second process named transactivation starts. Although this mechanism stimulates the transcription of some inhibitory genes, it mainly mediates the activation of gluconeogenesis, insulin resistance, skin atrophy, and the inhibition of bone formation, all well-known adverse effects of GCs [7,8,9].

The use of low doses of prednisone (≤7.5 mg/day) is associated with a progressive saturation up to 50% of the cGR. At medium doses (>7.5 mg/day to 30 mg/day of prednisone), the receptor becomes progressively saturated from 50 to close to 100%, keeping a less lineal relation with the daily dose. It is estimated that the almost complete saturation of cGR occurs at approximately 30–40 mg/day of prednisone. At higher doses, up to 100 mg/day, the predominant effect is transactivation, and therefore the occurrence of unwanted effects with no major increase in anti-inflammatory actions [10,11,12]. This pharmacodynamic behavior is the basis of the new GC dosage schemes [12].

### 1.2. What Is the Non-Genomic Way and How Does It Get Activated?

A second mechanism of action of GCs, the non-genomic pathway, acts by modulating inflammatory and immune cells by three molecular mechanisms independent from nuclear interactions. First, the GC-cGR complex directly blocks the activation of phospholipase A2 and thus the production of arachidonic acid by a transcription-independent mechanism. Second, the activation of the membrane-bound GR (mGR) leads to the reduction of lymphocyte activity via the p38 MAP kinase. Third, nonspecific interactions with the cellular membranes of immune cells result in the inhibition of ATP production and thus decrease cell activity [13]. In addition, mGR activation also modifies gene expression, so priming the immune cells for the upcoming genomic effects [13]. These non-genomic mechanisms are characterized by a rapid onset of action (less than 15 min) because they do not need time for translation to the nucleus and modulation of gene transcription. 

The activation of the non-genomic pathway starts at doses >100 mg/day of prednisone or equivalent. This pathway is especially sensitive to methylprednisolone (MP) and dexamethasone, which have non-genomic effects up to five times more potent than genomic ones [8].

Non-genomic effects are responsible for the efficacy of pulse therapy with GCs at doses over 125 mg of MP [14]. Work by the group of Buttgeterit et al. has shown the relative anti-inflammatory potency of different GCs by the non-genomic method, based on the effects on respiration, protein synthesis, and Na+−K+-ATPase and Ca2+−ATPase in concanavalin A-stimulated rat thymocytes [15]. MP and dexamethasone show the highest non-genomic-mediated potency (Table 1). The potency of treatment with MP also allows a faster tapering of oral prednisone and therefore, a reduction in the cumulative dose of GC [14,16,17]. A summary of the genomic and non-genomic GC effects is shown in Table 2.

### 1.3. Should High Doses of Prednisonse Be Still Considered the Standard Starting Dose?

**Figure 1:**
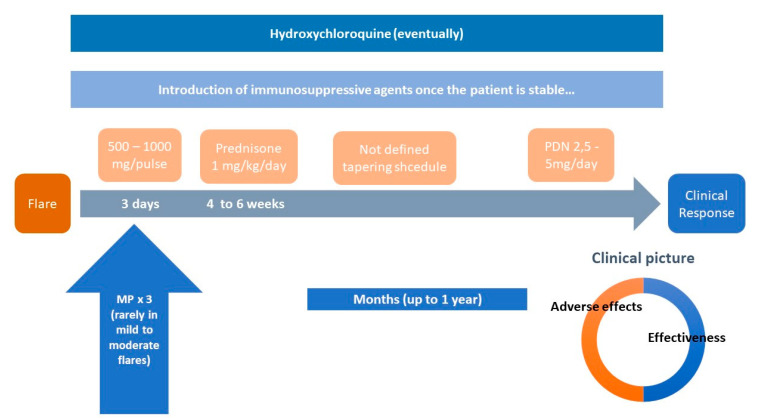
The “classical paradigm” in SLE therapy. Note: PDN: prednisone; MP: methylprednisolone pulses; proportions showed in “clinical picture” are merely illustrative.

The “classical” standard 1 mg/kg/day prednisone dose is not supported by either basic pharmacology or clinical evidence (Figure 1) [19,20]. It is unlikely that anti-inflammatory effects increase significantly after prednisone doses have reached 30–40 mg/day, since such doses already result in a saturation of almost 100% of the genomic pathway [12,19]. Recent data suggest that higher initial doses of prednisone are associated with higher cumulative doses [21] with the well proven result of increasing damage accrual [1,22,23,24,25].

**Figure 2:**
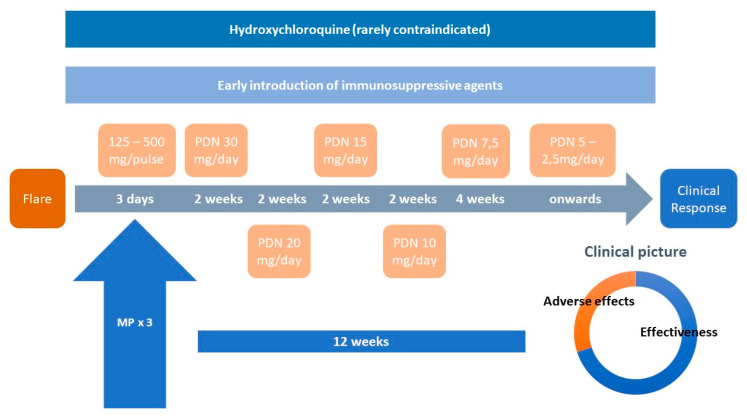
The “new paradigm” in SLE therapy. Note: PDN: prednisone; MP: methylprednisolone pulses; proportions showed in “clinical picture” are merely illustrative.

Instead, the combination of MP pulses followed by doses of prednisone up to 30 mg/day, depending on severity, is more effective, more rapid, and safer than the use of the “classical” 1 mg/kg/day (Figure 2). Several studies support this view. A European multicenter randomized, controlled study compared standard dose (1 mg/kg/day, *n* = 42) and reduced dose prednisone groups (0.5 mg/kg/day; *n* = 39), both associated with mycophenolic acid, during the induction phase of class III-IV lupus nephritis (LN). The complete remission rates at week 24 were similar for both groups, with fewer infections in the reduced prednisone dose group [26].

In an observational study of patients with class III-IV-V LN from the *Lupus-Cruces* (CC; *n* = 29) and the *Lupus-Bordeaux* cohorts (BC; *n* = 44), the number of pulses of MP per patient (9.3 vs. 2.3), but not the cumulative dose, and the proportion of patients on hydroxychloroquine (100% vs. 63%) were higher in the CC. The maximum doses of prednisone (21 vs. 42 mg/day), the number of weeks until 5 mg/day (12 vs. 22), and the mean doses at six months (8.3 vs. 21 mg/day) were all lower in the CC. Complete renal remission rates were significantly higher in the CC at six (69% vs. 30) and 12 months (86% vs. 43%) [27]. Of note, the number (not the total dose) of MP pulses was the only independent therapeutic predictor of achieving complete remission and of reducing GCs-related side effects [27].

The AURA–LV was a 48-week randomized clinical trial comparing the efficacy in the treatment of LN of two doses of voclosporin or placebo added to mycophenolate mofetil. All patients received a maximum initial dose of 25 mg/day of prednisone with tapering to 5 mg in 8 weeks and to 2.5 mg in 12 weeks. Remission rates at 48 weeks were 49.45% and 39.8% in both voclosporin groups [28]. Even patients in the control arm of the AURA trial had remission rates higher than those in previous studies such as ALMS [29] and LUNAR [30,31].

The “Rituxilup” schedule, which consisted of rituximab and MP, followed by maintenance treatment with mycophenolate mofetil and no oral steroids, resulted in 72% of patients with LN class III, IV, or V eventually achieving complete remission within a median period of 36 weeks [32].

In patients presenting with an SLEDAI score ≥6 (those with severe LN excluded), initial therapy with doses of prednisone ≤30 mg/day resulted in a similar decrease in SLEDAI scores at one year and reduced damage at five years compared with initial doses >30 mg/day. It must be noted that, in order to reduce prednisone doses, hydroxychloroquine was used in 100% vs. 33% of patients and MP pulses in 34% vs. 10%, respectively [1].

Thus, current evidence, based on large observational cohorts and a few clinical trials, supports the idea that low-medium initial doses of prednisone (i.e., ≤30 mg/day) are at least as effective as high-dose schemes and with a better safety profile [31,33]. MP pulses offer additional potency and allow the use of lower doses of prednisone. Of note, no studies of similar quality have ever shown the superiority of high-dose prednisone regimes.

### 1.4. Are 1000 mg MP Pulses More Effective than Lower Doses?

In a study from 1987 including 21 patients with active SLE with severe manifestations refractory to other treatments, patients were either treated with intravenous MP 100 mg/day or 1000 mg/day for three days. The study did not find significant differences between the two groups [34]. In addition, a retrospective study by Badsha et al., 2002 reported that 1500 mg of MP given throughout three days were equally effective in controlling the disease and associated with fewer serious infections than 3000 to 5000 mg in the same period [35]. The same authors carried out a prospective study comparing the use of 500 mg/day of methylprednisolone for three days with a historical cohort given higher doses and obtained the same results [36].

In the *Lupus-Cruces* cohort, the use of intravenous MP pulses, in all cases between 125 and 500 mg/day for three consecutive days, were not associated with long-term damage accrual [17]. In the previously mentioned study of patients with class III-IV-V LN from the CC and the BC, the number of intravenous MP pulses, rather than the total dose, was an independent predictor of complete response and of reduced GC-related toxicity. CC patients were treated with three consecutive 250–500 mg intravenous pulses and then with additional 125 mg pulses every two weeks before each intravenous dose of cyclophosphamide [27].

In 2018, Danza et al. compared the efficacy and rates of infections among patients with several autoimmune conditions, including SLE, treated with MP pulses, for a total dose over three days ≤1500 mg, <1500 to ≤3000 mg and >3000 mg [19]. No differences among the different doses were seen in patients achieving complete response, partial response, or no response. No patients in the ≤1500 mg group suffered infections, vs. 9.1% in the high dose group.

### 1.5. Should Pulses of MP Be Reserved for Life Threatening Flares?

It is well assumed that MP pulses, due to their higher potency and faster mechanism of action compared to oral prednisone, are indicated in those patients with severe manifestations of SLE in whom a rapid effect is necessary [4,14]. However, they might not be limited to this clinical scenario. The rapidity and potency of action make MP pulses at doses 125–250 mg/day for three days is ideal to deal with many moderate lupus flares, like arthritis, skin rashes, and pericarditis, and also for recurrent or non-responding mild flares. Thus, MP pulses may contribute to avoid high-dose prednisone and to promote a faster tapering, reducing the cumulative GC dose and thus the short and long-term side effects of oral prednisone [4,33].

### 1.6. Can GC-Related Damage Be Avoided without Reducing Efficacy?

There are several factors that contribute to organ damage accrual in patients with SLE. Among them, the role of GCs has proven fundamental. In 2003, Gladman et al. categorized damage as definite, probable and not related to GCs [3] and found that the latter increased over disease course, being the most frequent in latter stages of SLE. Joo et al. have also found that patients with LN have more damage associated than non-associated to GCs [37]. In the Hopkins cohort, GC-related irreversible damage has been shown to depend on the cumulative dose of prednisone [38]. Compared to patients who did not receive prednisone, the risk of accruing damage increased 1.2 times if they had received a cumulative dose of 180 mg per month, and twice for a cumulative dose >540 mg per month [23]. On the other hand, the use of MP pulses has not been associated with damage accrual in large series [17,38].

A possible reason explaining this is the different toxicity associated with the activation of the genomic (increasing toxicity in parallel with activation) and the non-genomic ways (free of genomic transactivation-related toxicity). Therefore, the use of MP pulses (activating non-genomic mechanisms), rather than prednisone doses >30 mg/day (fully activating the genomic way), for inducing rapid remission, followed by maintenance doses of prednisone ≤5 mg/day (activating less than 25% of the genomic way) may be a good approach to minimize GC-related side effects. In addition, hydroxychloroquine helps spare prednisone and, by itself, also prevents damage accrual [39]. Ruiz-Arruza et al found that the use of this treatment scheme achieved the same efficacy, with no increase in SLE-related damage and less global, cardiovascular and GC-related damage [33].

Whilst the eventual discontinuation of GC is the ultimate goal, a recent monocentric, 12-month, superiority, open label, randomized controlled trial compared the efficacy to prevent flares of maintenance versus withdrawal of 5 mg/day prednisone in patients with clinically quiescent SLE. The study found that maintenance therapy with 5 mg/day of prednisone prevents relapses, with no worsening of damage and no GC toxicity observed during the follow-up period [40]. Therefore, long-term therapy with low-dose GC may be necessary in a number of patients with SLE. This could contrast with the results of the aforementioned Rituxilup study [32], in which no oral GCs were used during the induction phase in patients with LN. However, the clinical setting (maintenance and induction) is different, and no data were given regarding how many patients in this study eventually needed GCs for other manifestations of SLE.

### 1.7. How Can the Risk of Infections Be Reduced during GC Treatment?

Infections represent one of the most important causes of morbidity and mortality in patients with SLE [41]. The factors predisposing lupus patients to infection are not only disease activity and the malfunction of the immune system, but also the use of immunosuppressive drugs, particularly GCs [41,42]. GCs suppress the production of inflammatory cytokines, the microbicidal activity of activated macrophages, the adhesion of neutrophils to endothelial cells, the release of lysosomal enzymes, the respiratory burst, and the chemotaxis. In addition, they cause marked lymphopenia in all lymphocyte subpopulations, inhibit the activation of T cells and have immunosuppressive effects on the maturation and function of dendritic cells, responsible for triggering the adaptive immune response [43]. Although these effects increase with the dose and the duration of treatment, the risk of infection is already high at maintained doses of 7.5 mg/day. Indeed, the chance of suffering a severe infection increases by 12% for each mg/day of prednisone [2].

According to the data obtained from the Spanish Registry of *Systemic Lupus Erythematosus* (RELES), 6.4% of patients had a documented episode of major infection during the first year of follow-up and 5.67% during the second. Mean prednisone doses >30 mg/day during the first month and >7.5 mg/day during the first year independently predicted major infections within the first and the second year of follow-up, respectively [44]. Regarding MP pulses, the use of 1000 mg/day for three days has been associated with an increase in infections compared with 500 mg/day [35].

A number of prophylactic measures, such as the administration of vaccines, can be recommended in SLE, and the use of hydroxychloroquine is also protective against infections [45]. However, a retrospective, new-user study including 3030 SLE patients found that the rate of severe infections in patients on prednisone >15 mg/day was high and not influenced by antimalarials use [22]. Thus, the use of maintenance doses of prednisone not exceeding 5 mg/day with pulses of 500 mg of MP instead of 1000 mg is probably a good way to reduce the infectious complications in lupus patients.

### 1.8. How Should GC Therapy Be Managed during Pregnancy?

Since the therapeutic possibilities are lower in pregnant women, GCs are one of main therapeutic resources during gestation in case of lupus flares [46]. Indeed, their potent anti-inflammatory effect seems not to be accompanied by any significant teratogenicity.

The choice of GC will depend on whether our goal is to treat the mother or the fetus. According to the most recent EULAR and BSR guidelines, in the first case, the use of non-fluorinated GCs such as prednisone or MP will be of choice, while in the second case, the treatment will be with fluorinated GCs, such as betamethasone or dexamethasone [46,47]. This is due to the presence of the placental 11-beta dehydrogenase enzyme that converts non-fluorinated GCs into relatively inactive forms, with less than 10% of the drug reaching the fetal circulation [48].

The adverse effects observed are similar to those occurring outside pregnancy, however, hypertension, preeclampsia, insulin resistance, infections and premature rupture of membranes represent additional serious problems during gestation. For this reason, prednisone is recommended at doses not exceeding 20 mg/day for the treatment of severe manifestations of the disease, and 7.5 mg/day for minor manifestations, with rapid tapering in both cases to maintenance doses ≤5 mg/day. If necessary, in moderate-severe flares, intravenous pulses of MP 125–500 mg can be safely used [49].

During the lactation, the amount of prednisone found in breast milk is very low, although it is advisable to delay breastfeeding until four hours after taking doses greater than 50 mg/day (which are not recommended, anyhow) [46].

In women undergoing corticotherapy during pregnancy or lactation, adequate supplementation with calcium 1000 mg/day and vitamin D 800 IU/day is recommended for the prevention of GC-induced osteoporosis [50].

### 1.9. What Are the Current Recommendations?

The current recommendations are, once a SLE flare is diagnosed, to achieve remission or low disease activity as soon as possible, and then prevent new flares [4,51,52]. An intensification of the immunosuppressive regimen is universally recommended for this purpose. Although there is no universal agreement regarding the definition of low dose of GCs, most authors accept doses ≤7.5–5 mg/day of prednisone or equivalent [52,53]. Since no consensus about tapering has been established, such doses can be reached within variable periods ranging from four weeks to up to 12 months.

Table 3 shows how starting doses of GCs vary among the different guidelines, from 0.3 to 2 mg/kg/day for patients with severe, renal or extra-renal involvement [51,52,54,55,56], or whenever the administration of MP pulses during induction therapy is not possible [54]. Even though the recent 2019 EULAR recommendations do not provide a specific tapering scheme, they recommend avoiding an initial dose of 1 mg/kg/day of prednisone and highlight the importance of using early MP pulses and immunosuppressants in order to spare oral GCs [4], with some significant recommendations: in cases of mild to moderate disease, start with prednisone doses ≤0.5 mg/kg/day, with “gradual tapering”; in cases of severe or organ-threatening disease, MP pulses (250–1000 mg/day) for 1–3 days are suggested, followed by prednisone 0.5–0.7 mg/kg/day “with tapering” [4]. Recommended MP doses vary from 250 mg/day to 1000 mg/day for 3 days when a flare is diagnosed [54]. Again, there is no agreement on its use during induction, often being reserved for severely active patients who do not achieve a sufficient response after initial high doses of prednisone [54,55].

### 1.10. What Is our Proposed “Standard of Care” for GC Use?

**Figure 3:**
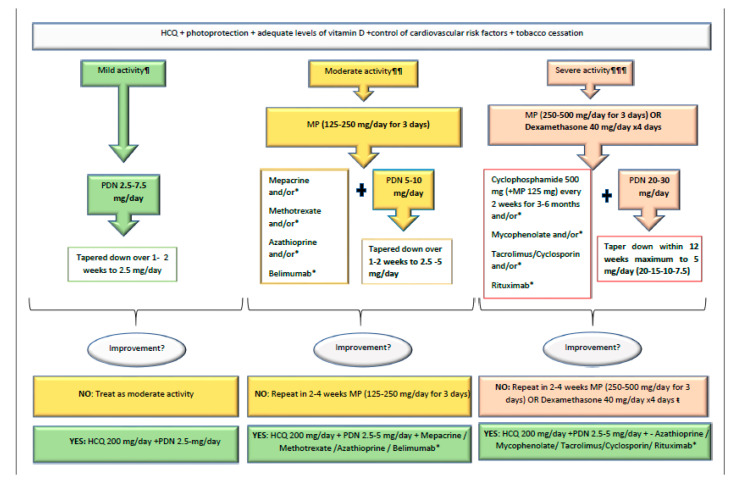
The Lupus-Cruces protocol for the treatment of SLE according to severity. Note: HCQ: hydroxychloroquine; PDN: prednisone; MP: methylprednisolone pulses; ¶ Polyarthralgia, small joint monooligoarthritis, limited skin lesions; ¶¶ Polyarthritis, moderate thrombocytopenia (20,000–50,000/mm^3^), haemolytic anaemia with a low rate of haemolysis, widespread skin lupus lesions, non-severe pericardial effusion/pericarditis, pleural effusion; ¶¶¶ Lupus nephritis, pneumonitis, severe thrombocytopenia (<20,000/mm^3^), haemolytic anaemia with a high rate of haemolysis, severe pericardial effusion, refractory pleural effusion, severe neuropsychiatric manifestations; * depending on specific organ involvement.

We advocate for a new paradigm in the use of GCs in SLE. The old, vague expression “the shortest time and at the lowest dose possible” is not enough, since many doctors feel that a rapid decrease in the dose of prednisone can precipitate a flare or a situation of adrenal insufficiency (Figure 1). Nowadays, there is enough evidence to support the use of doses of prednisone ≤30 mg/day in most severe flares, equally effective than higher doses, with a much better safety profile, both in the short and in the long term. MP pulses should not be limited life-threatening situations since they contribute to rapid disease control while sparing oral GCs. Pulses can be used in a wide range of doses, from 125 mg to 500 mg, depending on the severity of flares (Figure 2 and Figure 3). Using this scheme, along with hydroxychloroquine and early immunosuppressive therapy, it is possible to accomplish a quick tapering of prednisone, and therefore to achieve maintenance doses ≤5 mg/day within a maximum period of 12 weeks [57,58].

## 2. Conclusions

- Glucocorticoids may act by genomic and non-genomic pathways. The second way is faster and non-related to chronic damage.
- The classic glucocorticoid dose of 1 mg/kg/day is not evidence-supported and has a well-known range of serious adverse effects
- Recruiting the non-genomic pathway by methylprednisolone pulses followed by a reduced dose scheme of prednisone may avoid adverse effect and chronic damage
- Immunosuppressive agents should be early introduced in the treatment of moderate-severe SLE to spare glucocorticoids
- Prednisone maintenance doses ≤5 mg/day should be ideally achieved in no more than 12 weeks.
- Hydroxychloroquine is mandatory in SLE treatment, except in the exceptional cases with contraindications.

## Figures and Tables

**Table 1:** Anti-inflammatory potency exerted by genomic/non-genomic ways of the different glucocorticoids [15,18].

Glucocorticoid	Anti-Inflammatory Effect (Genomic Way)	Anti-Inflammatory Effect (Non-Genomic Way)
Cortisol/hydrocortisone	1	Low
Prednisone/prednisolone	4	4
Methylprednisolone	5	10–15
Dexamethasone	20–30	20
Betamethasone	20–30	<4

**Table 2:** Summary of the mechanisms of action of glucocorticoids.

	Genomic Pathway	Non-Genomic Pathway
Cells targeted	All the organism	Inflammatory cells
Mechanism of action	Genomic modulation	Membrane receptor and intracellular inflammatory pathways
Start of action	~4 to 6 h	~15 min
Saturation dose of the immunosuppressive – anti-inflammatory effects	~100% at 30 to 40 mg/day of prednisone-equivalent	Unknown
Minimum effective dose	2.5 to 5 mg/day of prednisone-equivalent	Over 100 mg of prednisone-equivalent
Maximum effective doses that minimize adverse effects	30 to 40 mg/day of prednisone-equivalent (for trans-repression)	500/day mg of methylprednisolone
Damage accrual with cumulative doses	Proven	Not proven
Glucocorticoids acting by this way	All	Mainly methylprednisolone and dexamethasone

**Table 3:** Recommended doses of glucocorticoids in recent lupus guidelines.

Guideline	Methodology	Clinical Setting	Pulses Recommended?	Dose of Prednisone Recommended?	Tapering Scheme?	Maintenance Dose?
ACR (2012) [54]	Opinions of highly-qualified experts.	LN III–IV	YES. 500–1000 mg/day methyl-prednisolone for 1–3 days	YES. 0.5–1 mg/kg/day or 1 mg/kg/day if crescents seen	NO. Only “a few weeks”	NO Only “to lowest effective dose”
		LN V	NO	YES. 0.5 mg/kg/day	NO. Maintain initial dose by for 6 months	
EULAR/ERA-EDTA (2012) [55]	A modified Delphi method was used to compile questions, elicit expert opinions and reach consensus	LN III–IV	YES. 500–750 mg/day methyl-prednisolone for 1–3 days	YES. 0.5 mg/kg/day	YES. Maintain initial dose by 4 weeks, reducing to ≤10 mg/day by 4–6 months.	YES. ≤10 mg/day
		LN II	NO	YES. If proteinuria >1 g/24 h: 0.25–0.5 mg/kg/day		
BSR (2018) [51]	Evidence-based guidelines, supplemented as necessary with expert opinion and consensus agreement.	Mild activity flare	NO	YES. ≤20 mg/day	NO. Only maintain initial dose by 1–2 weeks	YES. ≤7.5 mg/day
		Moderate activity flare	YES. ≤250 mg/day methyl-prednisolone for 1–3 days	YES. ≤0.5 mg/kg/day	NO	YES. ≤7.5 mg/day
		Severe activity flare:	YES 500 mg/day methyl-prednisolone for 1–3 days	YES. ≤0.75–1 mg/kg/day or ≤0.5 mg/kg/day with pulses	NO	YES. ≤7.5 mg/day
EULAR (2019) [4]	Delphi method, to form questions, elicit expert opinions and reach consensus.	Mild-moderate flare	NO	YES. ≤0.5 mg/kg/day	NO. Only gradual tapering	YES. ≤7.5 mg/day
		Severe/organ-threatening disease:	YES. “Consider” 250–1000 mg/day methyl-prednisolone for 1–3 days	YES. 0.5–0.7 mg/kg/day	NO. Only “gradual tapering”	YES. ≤7.5 mg/day.
GLADEL/PANLAR (2019) [52]	GRADE	LN	NO	YES. 1–2 mg/kg/maximum 60 mg/day	NO. “Regardless of manifestations of disease, should prescribed at the lowest doses and for the shortest period of weather”	YES. ≤7.5 mg/day.
		Diffuse alveolar haemorrhage	YES	NO		
EULAR/ERA-EDTA (2020) [56]	Delphi methodology. Task Force voted on their level of agreement with the formed statements.	LN III-IV	YES. Total dose 500–2500 mg, depending on disease severity.	YES. 0.3–0.5 mg/kg/day	YES. 0.3–0.5 mg/kg/day for up to 4 weeks. Tapered to ≤7.5 mg/day by 3 to 6 months. Gradual withdrawal of treatment (glucocorticoids first, then immunosuppressive)	YES. ≤7.5 mg/day
		LN V		YES. 20 mg/day	YES. Tapered to ≤5 mg/day by 3 months	YES. ≤5 mg/day

ACR: American College of Rheumatology. EULAR: European League Against Rheumatism. ERA-EDTA: European Renal Association-European Dialysis and Transplant Association. BSR: British Society of Rheumatology. LN: Lupus nephritis. GLADEL: Grupo Latino-Americano de Estudio del Lupus. PANLAR: Liga Panamericana de Asociaciones de Reumatología.
